# 

**Published:** 1874-01

**Authors:** 


### CONTENTS.

**CONTRIBUTIONS.**

| | PAGE. |
|---|---|
| The Esthetics of Surgery, BEN H. PRATT, A. M | 193 |
| Vital Relations, P. H. HAYES, M. D. | 195 |

**CLIPPINGS.**

| | |
|---|---|
| The Sand Club, | 197 |
| Pulse of Animals, | 204 |
| English Groom and Yankee, | 214 |
| New Mode of Treating Dyspepsia, | 218 |

**CORRESPONDENCE.**

| | |
|---|---|
| Letters and Answers to Correspondents, | 205 |

**EDITORIAL.**

| | PAGE. |
|---|---|
| Medical Items and News | 198 |
| Household Hints and Recipes | 207 |
| Close of Volume, | 211 |
| Staphyloma, | 211 |
| Wry Neck, | 211 |
| The Baby, | 212 |
| Our Joints, | 213 |
| Chit-Chat | 214 |
| Books and Periodicals | 218 |

